# Endovascular Biopsy of Vertebrobasilar Aneurysm in Patient With Polyarteritis Nodosa

**DOI:** 10.3389/fneur.2021.697105

**Published:** 2021-11-23

**Authors:** Kazim H. Narsinh, Kamileh Narsinh, David B. McCoy, Zhengda Sun, Cathra Halabi, Karl Meisel, Tarik Tihan, Krishna Chaganti, Matthew R. Amans, Van V. Halbach, Randall T. Higashida, Steven W. Hetts, Christopher F. Dowd, Ethan A. Winkler, Adib A. Abla, Tomasz J. Nowakowski, Daniel L. Cooke

**Affiliations:** ^1^Division of Interventional Neuroradiology, Department of Radiology and Biomedical Imaging, University of California, San Francisco, San Francisco, CA, United States; ^2^Division of Neurovascular Neurology, Department of Neurology, University of California, San Francisco, San Francisco, CA, United States; ^3^Weill Institute for Neuroscience, University of California, San Francisco, San Francisco, CA, United States; ^4^Division of Neuropathology, Department of Pathology, University of California, San Francisco, San Francisco, CA, United States; ^5^Division of Rheumatology, Department of Medicine, University of California, San Francisco, San Francisco, CA, United States; ^6^Cerebrovascular Disorders Program, Department of Neurological Surgery, University of California, San Francisco, San Francisco, CA, United States; ^7^Department of Anatomy, Chan Zuckerberg Biohub, University of California, San Francisco, San Francisco, CA, United States

**Keywords:** fusiform aneurysm, gene expression profile, single cell RNA sequencing (scRNA-seq), polyarteritis nodosa (PAN), endovascular biopsy

## Abstract

**Background and Purpose:** The management of unruptured intracranial aneurysms remains controversial. The decisions to treat are heavily informed by estimated risk of bleeding. However, these estimates are imprecise, and better methods for stratifying the risk or tailoring treatment strategy are badly needed. Here, we demonstrate an initial proof-of-principle concept for endovascular biopsy to identify the key molecular pathways and gene expression changes associated with aneurysm formation. We couple this technique with single cell RNA sequencing (scRNAseq) to develop a roadmap of the pathogenic changes of a dolichoectatic vertebrobasilar aneurysm in a patient with polyarteritis nodosa.

**Methods:** Endovascular biopsy and fluorescence activated cell sorting was used to isolate the viable endothelial cells (ECs) using the established techniques. A single cell RNA sequencing (scRNAseq) was then performed on 24 aneurysmal ECs and 23 patient-matched non-aneurysmal ECs. An integrated panel of bioinformatic tools was applied to determine the differential gene expression, enriched signaling pathways, and cell subpopulations hypothesized to drive disease pathogenesis.

**Results:** We identify a subset of 7 (29%) aneurysm-specific ECs with a distinct gene expression signature not found in the patient-matched control ECs. A gene set enrichment analysis identified these ECs to have increased the expression of genes regulating the leukocyte-endothelial cell adhesion, major histocompatibility complex (MHC) class I, T cell receptor recycling, tumor necrosis factor alpha (TNFα) response, and interferon gamma signaling. A histopathologic analysis of a different intracranial aneurysm that was later resected yielded a diagnosis of polyarteritis nodosa and positive staining for TNFα.

**Conclusions:** We demonstrate feasibility of applying scRNAseq to the endovascular biopsy samples and identify a subpopulation of ECs associated with cerebral aneurysm in polyarteritis nodosa. Endovascular biopsy may be a safe method for deriving insight into the disease pathogenesis and tailoring the personalized treatment approaches to intracranial aneurysms.

## Introduction

Intracranial aneurysms are a common cause of hemorrhagic stroke, resulting in severe morbidity and mortality worldwide (1). Vertebrobasilar aneurysms are particularly difficult to manage because of a morbid natural history (2) and the high rates of complication (3) or retreatment (4) with open or endovascular treatment approaches. Better tools are needed to determine patient-specific risk of aneurysm rupture and derive insight into their underlying pathogenesis. Interestingly, the anti-inflammatory medications have been suggested as protective against the aneurysm growth or rupture, although the aneurysm features that may dictate such a response to medication have not yet been elucidated (5–7). Transcriptomic classification of aneurysms may help unravel these variable drug responses. “Precision medicine” refers broadly to such treatment approaches that account for the patient-specific variability in genetics, environment, or lifestyle, in contrast to a “one-size-fits-all” approach. However, the precision medicine approaches to intracranial aneurysms are limited by a dearth of aneurysmal tissue, whose analysis may provide insight into the molecular pathways underlying pathogenesis (8–10). Obtaining aneurysm tissue antemortem has typically necessitated craniotomy with its attendant risks. Also, craniotomy is typically only performed when the surgical treatment is predetermined, making the results of the tissue analysis somewhat inconsequential in that the non-surgical treatment is already disfavored. Moreover, the aneurysm tissue obtained, when subjected to bulk immunohistochemical, transcriptomic, or proteomic analysis, the yields coarse information that may belie the contribution of rarer cell types to disease pathogenesis. The single cell RNA sequencing (scRNA-seq) has emerged as a powerful tool for elucidation of cellular heterogeneity and insight into disease pathogenesis. Indeed, the cellular resolution transcriptomics and epigenomics have revealed a heightened degree of cellular diversity in the cerebral vasculature beyond the canonical cell-type definitions (11). If a minimally invasive endovascular biopsy technique could yield cells amenable to scRNAseq, significant advances could be made in applying the precision medicine approaches to cerebrovascular disease. Namely, two chief hurdles could be overcome. First, obtaining cells using the conventional endovascular tools would obviate the difficulty and risk of obtaining cerebrovascular tissue with open surgery. Second, the cellular and molecular heterogeneity of intracranial aneurysms could be better analyzed using the single cell sequencing techniques rather than the pooling cells to achieve sufficient input material for an assay. Herein, we obtained the aneurysmal endothelial cells (ECs) using the image-guided, catheter-based, minimally-invasive endovascular biopsy technique, then performed scRNA-seq on the sample, and used bioinformatic tools to generate hypotheses regarding disease pathogenesis. This proof-of-concept study demonstrates the feasibility of studying the transcriptomic mechanisms that mediate the formation of aneurysms at cellular resolution, pushing the field of cerebrovascular disease toward individualized diagnostics and therapeutics.

## Materials and Methods

### Protocol Approval and Patient Consent

This study was approved by the UCSF Human Research Protection Program (IRB) and upheld the ethical principles of the Helsinki Declaration. An informed written consent for participation in the research study was provided by the patient and can be provided upon request from the authors.

### Endovascular Biopsy and Endothelial Cell Enrichment

**Figure 1:**
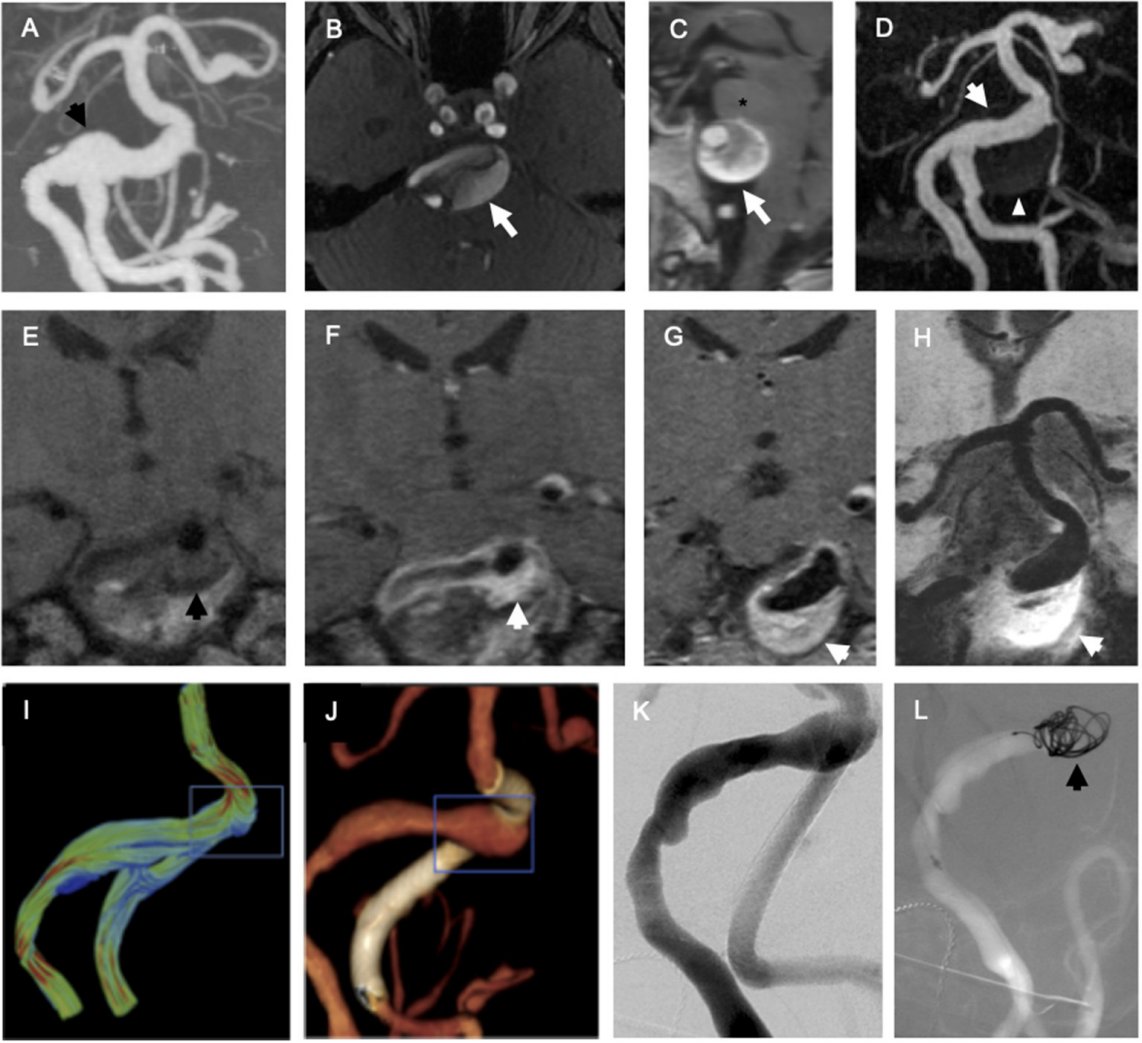
Endovascular biopsy of endothelial cells (ECs) from fusiform vertebrobasilar aneurysm. **(A)** Coronal maximum intensity projection (MIP) reformat of CT angiogram of the head focusing on the posterior intracranial circulation demonstrates fusiform vertebrobasilar aneurysm with focal dilation of the right vertebrobasilar junction (black arrow). **(B)** Axial time-of-flight magnetic resonance (MR) angiogram of the head demonstrates fusiform aneurysm involves the basilar artery (white arrow). **(C)** Sagittal T1-weighted MRI of the brain demonstrates fusiform aneurysm involving the basilar artery (white arrow) compresses the pons of the brainstem (asterisk). **(D)** Coronal MIP reformat of contrast-enhanced magnetic resonance angiography (MRA) demonstrates fusiform aneurysm involves the basilar artery (white arrow) and the partially thrombosed aneurysm wall is much larger than the white lumen (white arrowhead). Coronal T1-weighted MRI of the right vertebrobasilar junction before **(E)** and after **(F)** administration of gadolinium chelate contrast demonstrates intense enhancement of the aneurysm wall, suggestive of non-specific inflammation (black arrow = aneurysm wall before contrast and white arrow = aneurysm wall after contrast). **(G,H)** Hyperenhancement of the aneurysm wall (white arrow) is seen at the right vertebrobasilar junction on 0.6 mm slice thickness **(G)** and 10 mm minIP reformat **(H)** images. **(I)** A computational fluid dynamic model derived from angiographic data demonstrates high velocity vectors at the superior aspect of the vertebrobasilar junction (blue box). **(J)** Three-dimensional volume-rendered reformat of flat panel CT angiogram demonstrates focal dilation of the right vertebrobasilar junction (blue box) and flow-diverting stent in the left intradural vertebral artery (white). **(K)** Planar right vertebral arteriogram in left anterior oblique projection. **(L)** Roadmap right vertebral arteriogram in the same projection shows a platinum coil (black arrow) in contact with the endothelium of the right vertebrobasilar junction during endovascular biopsy.

For biopsy of the intracranial aneurysm, a platinum coil attached to a pusher wire (Target 360, Stryker, Salt Lake City, UT, USA) was advanced through a microcatheter in the right vertebral artery to make contact with the endothelium of the aneurysm at the right vertebrobasilar junction (Figure 1L). The coil did not contact non-aneurysmal endothelium. The coil was placed in dissociation buffer in a 50 ml conical tube, agitated, centrifuged, incubated in erythrocyte lysis buffer, then resuspended in fluorescence-activated cell sorting (FACS) buffer. For the non-aneurysmal peripheral control cells, a 0.035″ Bentson guidewire was advanced through a 6 Fr sheath in the right common femoral artery until it made contact with the endothelium of the right common femoral artery and right external iliac artery. After removing the wire from the body, the distal 7 cm was cut and placed in the dissociation buffer in a 50 ml conical tube, and then, processed in the same fashion as the aneurysm sample. After incubation with 4′,6-diamidino-2-phenylindole (DAPI), anti-CD31-, and anti-CD34-antibodies, the cell suspensions underwent FACS on an Aria II machine (BD Biosciences, San Jose, CA, USA). The cells positive for DAPI were deemed non-viable and excluded. The ECs negative for DAPI and double positive for CD31 and CD34 were sorted and individually loaded into a 48-well plate.

### Single Cell RNA-Sequencing

The cDNA libraries were prepared using the Smart-seq2 protocol as previously described (12–14) on a Fluidigm C1 system (Fluidigm, South San Francisco, CA, USA) and sequenced on a HiSeq2500 machine (Illumina, San Diego, CA, USA).

### scRNAseq Data Analysis

A CLC Genomics Workbench (Qiagen, Redwood City, CA, USA) was used for the alignment and normalization to calculate read per kilobase per million mapped reads (RPKM). The mapping was performed against human reference genome GRCh37. The quality control measures and single cell gene expression profiles were analyzed using the Seurat (15) and Bioconductor (16) packages in R. After filtering the cells with >10% mitochondrial RNA and regressing biological covariates (ribosomal contents and cell cycle effects), the *FindVariableFeatures* procedure in Seurat was used to identify a subset of 200 highly variable genes, which were used for the principal component analysis (PCA) and uniform manifold approximation and projection (UMAP). Complementary to this analysis, an unsupervised clustering algorithm in SC3 (17) was used to estimate the optimal number of clusters (*k* parameter) using a random matrix theory-based method (*sc3_estimate_k* function). The differentially expressed genes (DEGs) were identified using the Model-based Analysis of Single cell Transcriptomics (MAST) method implemented in the *FindAllMarkers* function within Seurat. The *P*-values were adjusted for multiple hypothesis testing using a false discovery rate (FDR) threshold, and the genes were considered differentially expressed when FDR-corrected *P* ≤ 0.05, and log_2_-transformed absolute fold-change ≥1. During the exploratory analysis of the pathways possibly implicated in the aneurysm phenotypes, the nominal *P*-values were used instead, always followed by the adjustment of *P*-values estimated from the pathway enrichment analysis. For an enrichment analysis, the reference gene sets from MSigDB hallmark processes were used, which were derived by the aggregation of multiple MSigDB gene sets that represented the well-defined biological states or processes (18). The gene set enrichment analysis (GSEA) framework 7 (19) was run in a pre-ranked mode, and an ordered gene list was produced per dataset by scoring each gene according to the following formula: *score* = −*log*_10_(*P*) × *sign*(*FC*), where *P* is the *P*-value estimated from the differential expression analysis, and FC is the corresponding fold-change value derived from comparisons between the two groups of cell populations. During the exploratory analysis of pathways possibly implicated in the aneurysm phenotypes, the nominal *P*-values were used, always followed by the adjustment of *P*-values estimated from the pathway enrichment analysis. In addition, 1,000 permutations of the gene sets were performed to calculate the gene-set FDR, and a cut-off of 0.05 was used to significantly define the enriched pathways. Only the biological process terms from the Gene Ontology (GO) were considered, and the GO terms were grouped when their kappa score was ≥0.4. The analysis and visualizations were produced in R (http://www.r-project.org/) using base functions and the packages ggplot2, circlize (20), pheatmap (https://github.com/raivokolde/pheatmap), and clueGO (21). Further details are provided in the Supplementary Methods.

### Data Availability Statement

Data used for the analysis are available from the corresponding author upon reasonable request.

## Results

### ECs From Vertebrobasilar Aneurysm Can Be Collected via Endovascular Biopsy Technique and Undergo Single Cell RNA Sequencing

A 56-year-old woman with an enlarging, partially thrombosed, vertebrobasilar aneurysm (Figure 1) underwent endovascular biopsy followed by flow-diversion. FACS-isolated ECs (CD31 and CD34 double positive, DAPI negative; 24 aneurysmal vertebrobasilar ECs, and 23 non-aneurysmal femoral artery ECs) underwent scRNAseq. Then, 46 of 47 ECs (98%) met quality control criteria for the analysis, yielding 1,946 ± 541 detected transcripts per cell (mean ± SD with RPKM ≥ 1) for analysis (see Supplementary Methods).

### DEGs in ECs Isolated From Vertebrobasilar Aneurysm vs. Peripheral Femoral Artery

We compared average expression of genes in the aneurysmal and peripheral ECs (Supplementary Figure 1). The differential gene expression analysis yielded 29 genes (Supplementary Figures 2, 3), although none of these genes met statistical significance criteria after multiple testing correction (Supplementary Table 1). The gene set analysis of all the detected transcripts demonstrated that many of the upregulated genes were involved in the innate immune system and class I MHC mediated antigen processing and presentation (ICAM3, AP1M1, IFI6, LMO7, and ATP6V0E2).

### A Single Cell Gene Expression Analysis Reveals Subpopulation of Aneurysmal ECs in a Different Transcriptional State

**Figure 2:**
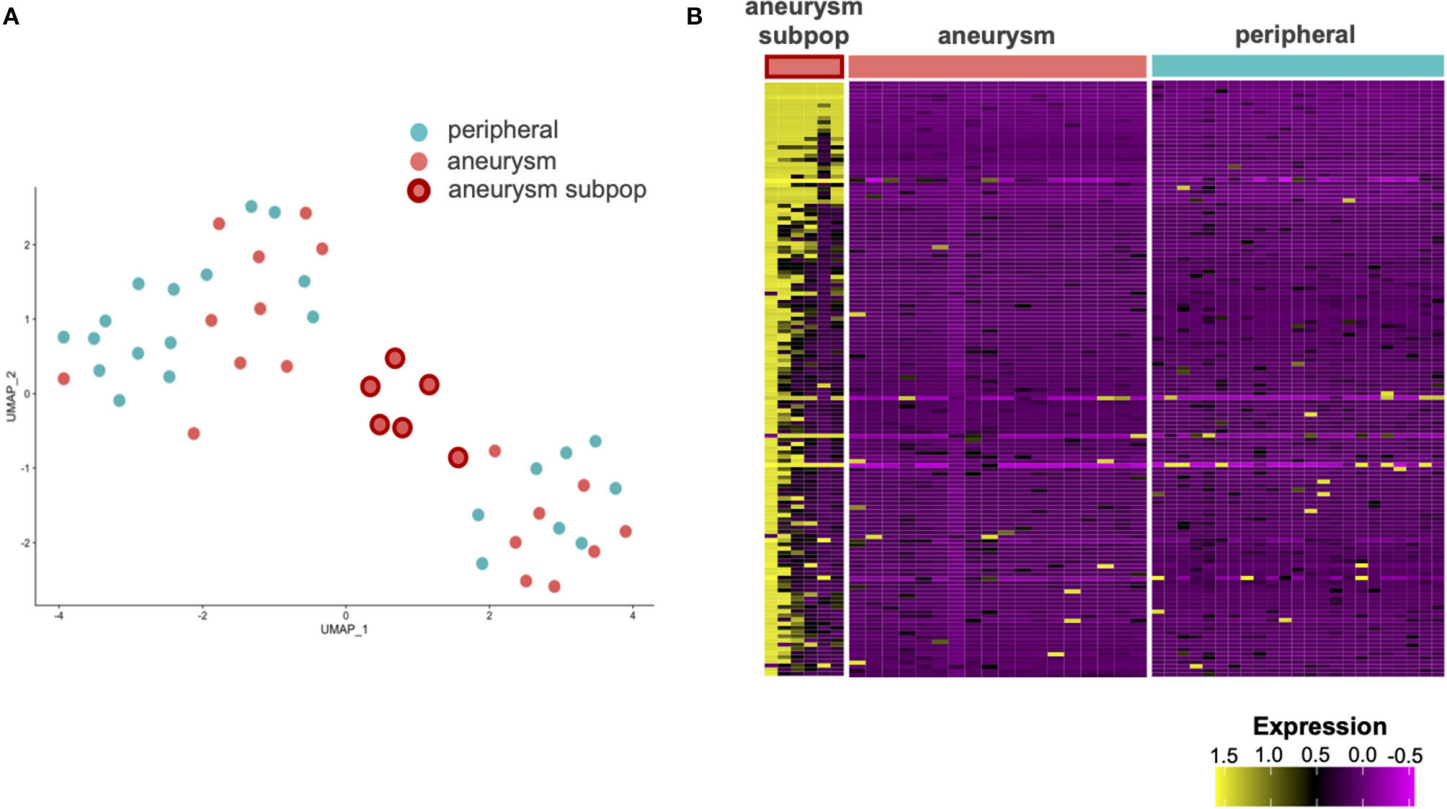
Single cell RNA sequencing (scRNAseq) of the ECs from aneurysm and femoral artery reveals differentially expressed genes (DEGs). **(A)** Uniform manifold approximation and projection (UMAP) analysis of scRNAseq results demonstrates a subpopulation of aneurysmal endothelial cells (orange with red border) in altered transcriptional state relative to the other aneurysmal ECs (orange) and non-aneurysmal peripheral femoral endothelial cells (turquoise). **(B)** A heatmap with the columns as cells and the rows as genes contained in the first principal component. A subpopulation of aneurysmal ECs (far left) has an altered gene expression profile relative to the remaining cells.

**Figure 3:**
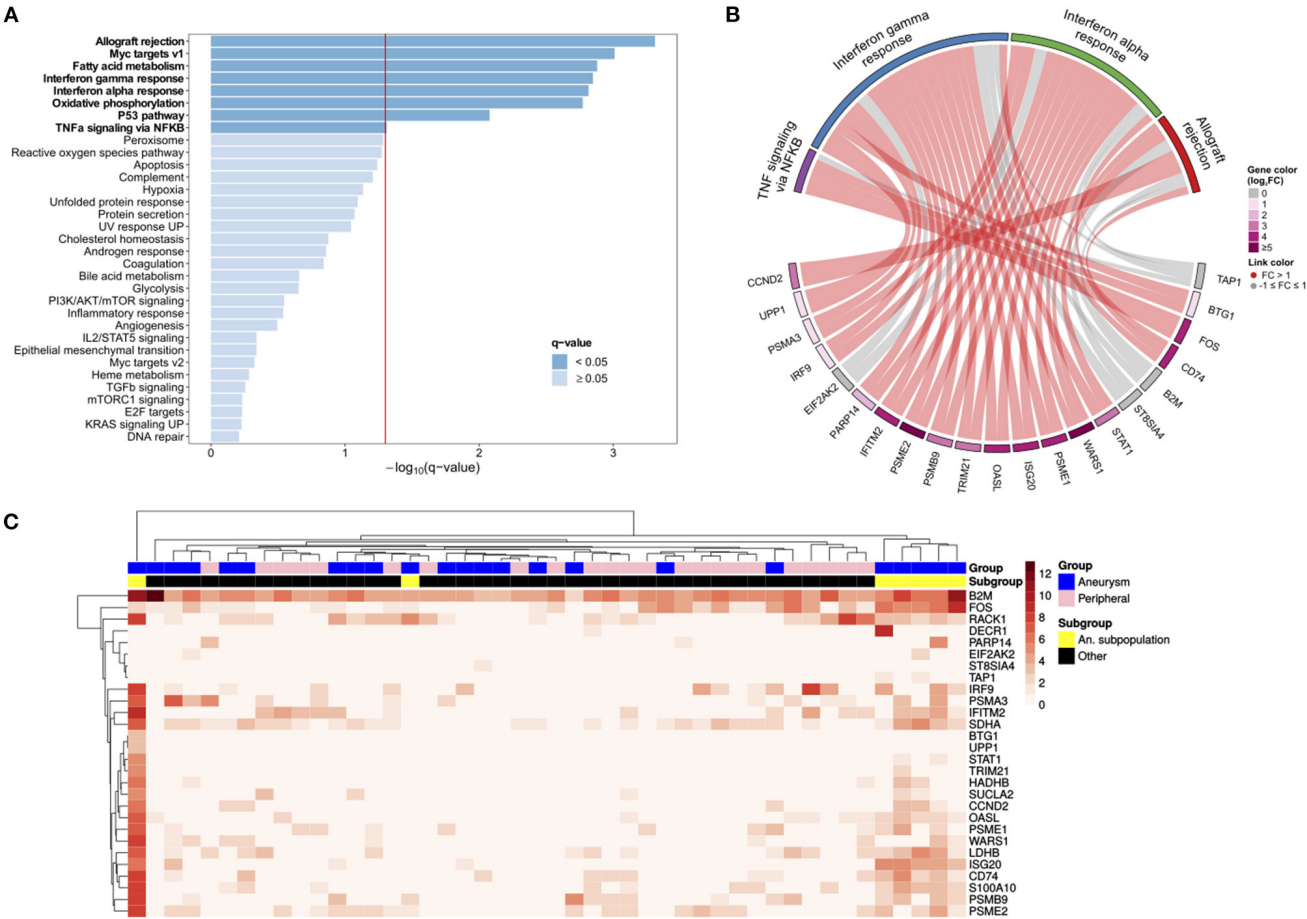
The scRNAseq analysis identifies aneurysm endothelial cell subpopulation with the upregulated immunologic pathways. **(A)** A Gene set enrichment analysis (GSEA) of aneurysm EC subpopulation compared with the remaining ECs demonstrates the enrichment of hallmark pathways for allograft rejection, Myc targets, interferon γ and α response, oxidative phosphorylation, the p53 pathway, and tumor necrosis factor alpha (TNFα) signaling *via* nuclear factor kappa-light-chain-enhancer of activated B (NFκB) (bold = statistically significant). **(B)** A chord diagram constructed using the significantly enriched hallmark pathways demonstrates relationship between individual genes (bottom) with pathways (top). **(C)** A heatmap with the cells as columns and the genes as rows subjected to hierarchical clustering shows a subpopulation of aneurysm ECs (far right; yellow) in a distinct transcriptional state.

Using the principal component analysis (PCA) and uniform manifold approximation and projection (UMAP) to analyze the single cell transcriptomes, we identify a subpopulation of ECs unique to the cerebral aneurysm with a distinct gene expression signature (Figure 2, Supplementary Figures 4, 5). To confirm, we used an unsupervised clustering algorithm to identify this distinct subpopulation, which expressed >100 DEGs involved in the tumor necrosis factor alpha (TNFα) signaling *via* nuclear factor kappa-light-chain-enhancer of activated B cells (NFκB), interferon alpha (IFNα), and interferon gamma (IFNγ) response, and allograft rejection (Figure 3, Supplementary Table 2). A separate posterior inferior cerebellar artery (PICA) aneurysm in the same patient was resected as part of an occipital artery to PICA bypass surgery, and underwent an immunohistochemical analysis, demonstrating aneurysm wall inflammation and strong positive staining for TNFα and NFκB (Supplementary Figure 6), suggesting the possibility of shared molecular pathogenesis between the biopsied and resected aneurysms.

### Functional Pathway and Network Analysis Identifies the Upregulated T-Cell Mediated Immune Pathways in Vertebrobasilar Aneurysm

**Figure 4:**
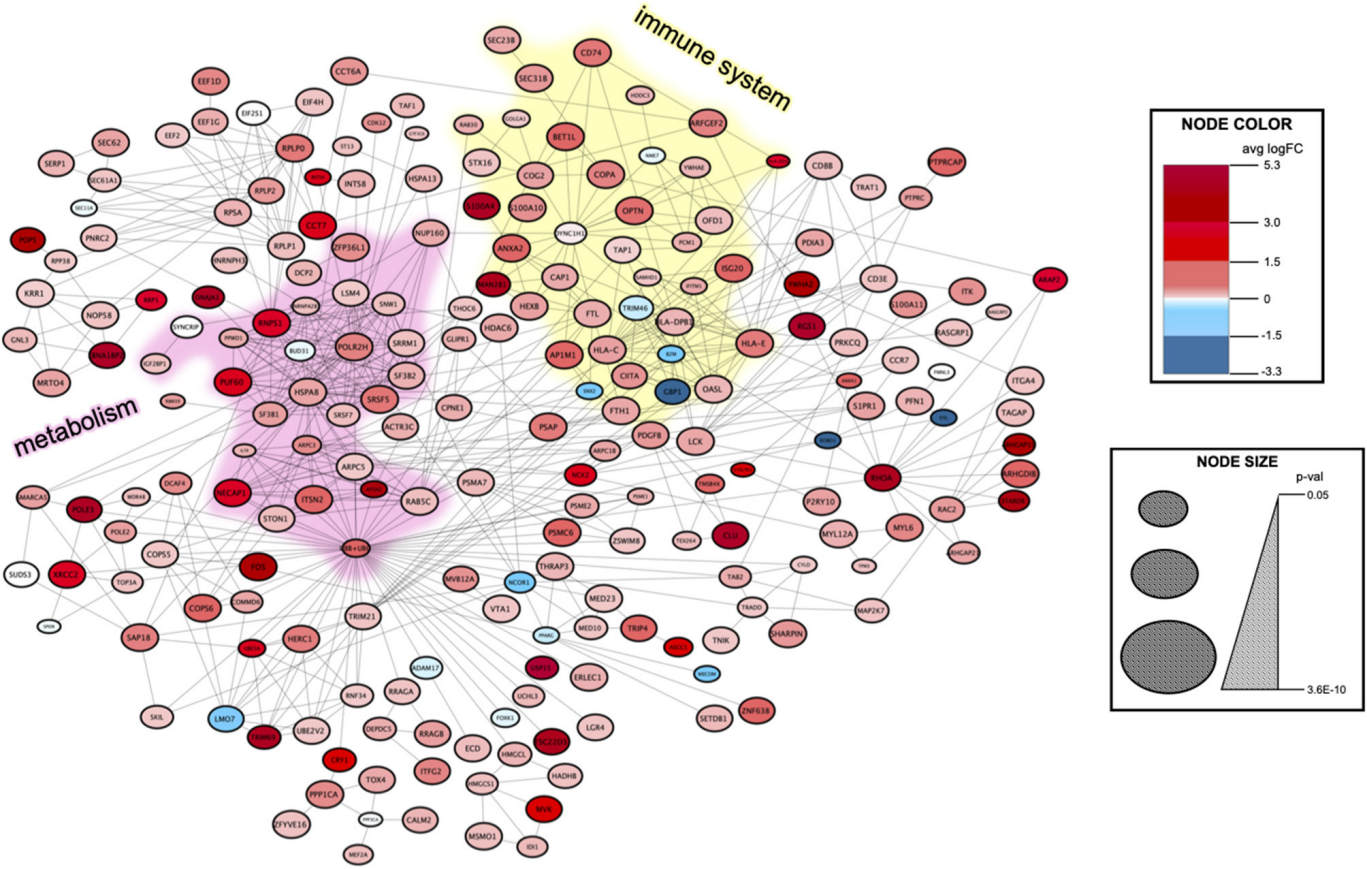
Protein–protein interaction network to identify the signal transduction pathways of interest in the aneurysm EC subpopulation. The DEGs between the aneurysm subpopulation and the remaining cells were identified using MAST, and then, fed into the STRING database (see Supplementary Methods). The resulting network contains 219 nodes with 630 edges. Clustering using the MCODE algorithm detects highly interconnected regions of the network. An immune system subnetwork is highlighted in yellow color and a metabolism subnetwork is highlighted in pink color (Supplementary Figure 7). Node color represents an average logarithmic fold change and node size corresponds to *p*-value (see legend at right).

**Figure 5:**
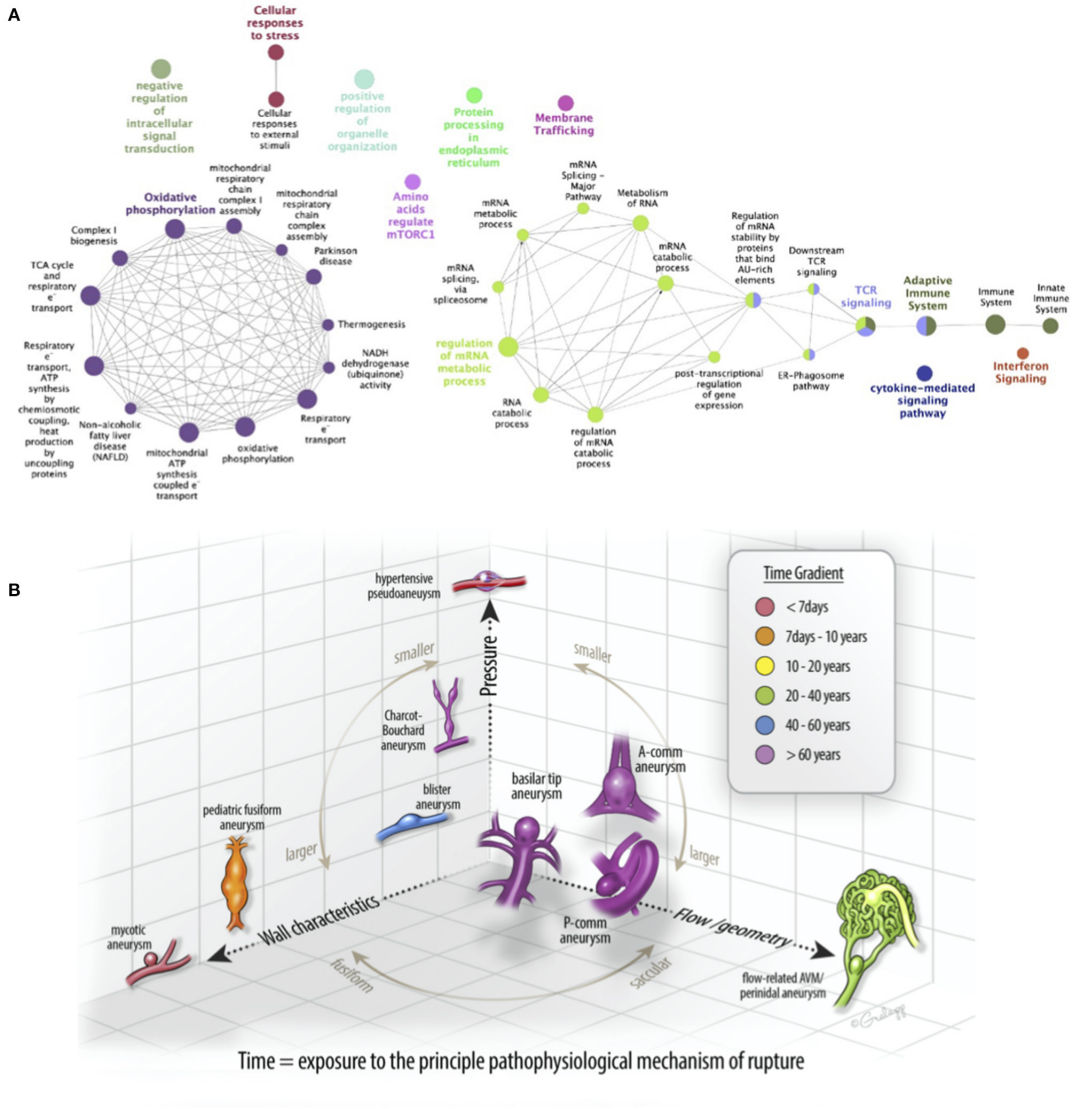
A patient-specific disease pathogenesis can be inferred from the endovascular biopsy results. **(A)** Significantly enriched pathways identified in the protein–protein interaction network were functionally grouped using clueGO to facilitate biologic interpretation. The innate immune system, adaptive immune system, interferon signaling, and cytokine-mediated signaling nodes to the far right are linked to mRNA regulation via T-cell receptor (TCR) signaling. **(B)** A schematic diagram of commonly diagnosed intracranial aneurysms in a three-dimensional space composed of principal pathophysiologic axes. *X*-axis corresponds to flow/geometry-driven pathology, best demonstrated in the flow-related arteriovenous malformation (AVM)-associated aneurysms. *Y*-axis corresponds to the wall characteristics, best demonstrated by “mycotic” or infectious aneurysms. *Z*-axis corresponds to pressure, best demonstrated by hypertensive or Charcot-Bouchard aneurysms. Most saccular aneurysms (e.g., A-comm, P-comm, and basilar tip) are driven pathophysiologically by a composite of these processes. Recognizing the diversity of the driving processes and elucidating them with gene expression profiling could advance our understanding of intracranial aneurysms.

To find the biological and signal transduction pathways of interest that defined the subpopulation of aneurysmal ECs, we constructed a protein–protein interaction network and performed functional enrichment after identifying the DEGs (see Supplementary Methods, Figure 4). The clustering analysis and functional enrichment revealed subnetworks enriched in the immune and metabolic pathways (Supplementary Figure 7). A network analysis of functionally grouped terms demonstrated that the adaptive immunity and T-cell receptor (TCR) signaling formed critical links between the immune system and transcriptional regulation of mRNA expression (Figure 5A). In addition, the upregulated genes were functionally enriched in the pathways responsible for oxidative phosphorylation.

## Discussion

Herein, we obtained the ECs from a vertebrobasilar aneurysm using an endovascular biopsy technique, and then, performed scRNA-seq on the obtained cells. By comparing the aneurysmal ECs with non-aneurysmal ECs obtained from the femoral artery, we were able to identify the DEGs that may drive disease pathogenesis. First, by comparing the average gene expression in the aneurysmal vs. peripheral ECs, we found the upregulation of five genes involved in the innate immune system and class I MHC mediated antigen processing and presentation. We subsequently analyzed the single cells and identified a subpopulation of cells in an altered transcriptional state. Comparing this subpopulation of cells to the remainder allowed identification of many more genes that were highly enriched in the TNFα signaling *via* NFκB, IFNα, and IFNγ response, and allograft rejection processes. An immunohistochemical analysis of a different aneurysm resected from the same patient demonstrated aneurysm wall inflammation with TNFα and NFκB involvement, and the patient was clinically diagnosed with polyarteritis nodosa.

Intracranial aneurysms are a common cause of hemorrhagic stroke, resulting in severe neurologic morbidity. The current management paradigms for unruptured intracranial aneurysms suffer from the two related limitations. First, they focus on observation vs. surgery without adequately considering the medical treatment as a reasonable alternative. For example, some observational studies have suggested that aspirin can decrease the rupture rate of intracranial aneurysms (22, 23), and at least two randomized clinical trials are ongoing (24, 25). Second, many ruptured intracranial aneurysms are small and would not meet risk stratification criteria for the treatment if they had been detected when unruptured (26, 27). Although this observation is related to the large number of small intracranial aneurysms detected, it underscores the need for better risk stratification criteria to determine which unruptured intracranial aneurysms are more likely to grow or hemorrhage. A method for gene expression profiling of aneurysmal tissue could provide richer, more granular data regarding the underlying pathophysiology of a particular aneurysm beyond the morphologic characteristics evaluated on vascular imaging, such as daughter sacs or blebs (28). Also, if prospective data supporting the medical treatments for aneurysms burgeon, it would be important to determine whether the administered treatment alters the molecular pathways driving the disease. A precision medicine approach to intracranial aneurysms would be greatly facilitated by an ability to biopsy aneurysmal tissue in a minimally invasive fashion. Such data could be used to determine the relative contribution of the principal pathophysiological mechanisms to aneurysm growth and rupture, such as pressure-mediated endothelial dysfunction, mural inflammation, or altered shear-stress, to a specific aneurysm in patient-specific fashion (Figure 5B). Understanding the relative contributions of these intrinsic and extrinsic mechanisms to aneurysm formation and rupture could better inform the disease management strategies, whether observational, surgical, or medical.

A polyarteritis nodosa is a systemic, autoimmune, necrotizing vasculitis affecting medium- and small-sized arteries, with poorly understood pathogenesis. A T-cell mediated immunity via antigen-specific crosslinking may be implicated (29, 30). The ECs regulate key aspects of the immune system, particularly the recruitment and activation of T cells (31, 32). Specifically, the ECs can activate effector/memory CD4 and CD8 lymphocytes and promote the expansion of one T cell subtype over another (helper, cytotoxic, or regulatory). Shimojima et al. have suggested that the defects in T-cell mediated immunity contribute to the development of polyarteritis nodosa, specifically citing a relatively low frequency of T_H_17 cells and a predominance of T_H_1 helper cells in the peripheral blood samples of the patients with polyarteritis nodosa compared with the patients with granulomatosis with polyangiitis or healthy controls (33). In addition, the ECs are shown to alter their metabolism from a quiescent to an active state in metabolic reprogramming during inflammation (34). Although our data cannot be generalized because we only analyzed one patient, our approach suggests myriad new targets for disease modification and directly links T cell dysregulation to the arterial wall pathology. These results, specific to this patient, differ from other studies of bulk RNA sequencing on intracranial aneurysm tissue that implicates B-lymphocytes in aneurysm rupture (35). Future studies will be needed to demonstrate the causation and disease modification with the immunomodulating therapies, such as with the anti-TNFα monoclonal antibody infliximab, that has already shown promise in the treatment of extracranial aneurysms for the patients with polyarteritis nodosa (36–38).

Our study has limitations. First, this study is based on a single patient. No conclusions can be drawn regarding the molecular definition of PAN and/or fusiform vertebrobasilar aneurysms based on this single case. Second, the control cells used were isolated from the femoral artery, which, in a systemic disease, such as polyarteritis nodosa, may also harbor the disease-specific gene expression profiles. Third, because of the limited intracranial aneurysm tissue available, the immunohistochemical confirmation of the hypotheses generated *in silico* are limited to inflammation in the aneurysm wall and positive staining for TNFα and NFκB. Our study has several strengths. This is a hypothesis-generating diagnostic study intended to demonstrate the feasibility of a personalized medicine approach to intracranial aneurysms. The biopsy material was obtained via minimally invasive, image-guided, endovascular approach without a craniotomy. The scRNAseq methodology enables whole-genome coverage at single cell resolution and requires less tissue as input material compared with the microarray or bulk RNA sequencing methods. This approach enables the discovery of novel cell subpopulations and genes that may be involved in the disease pathogenesis. The validation of these genes and pathways will require a larger number of samples and bench or animal modeling to gain confidence regarding the pathophysiologic mechanisms underlying these disorders. However, we wish to highlight the methodology employed here to permit these discoveries, however, limited. The human brain aneurysmal genetics has heretofore largely focused on the germline analyses using genome wide association studies (GWAS) or similar linkage methods (39). Although such efforts are of great value, they do not assay the aneurysm tissue specifically for the local gene expression alterations. Small series have described the gene expression profiles from the aneurysmal domes collected as part of clipping (35, 40, 41); however, this type of assay is not possible for those aneurysms not amenable to open surgical treatment (similar to most fusiform vertebrobasilar aneurysms). With this manuscript, we advance the concept of endovascular biopsy and scRNAseq as a next step toward a precision medicine approach to neurovascular disease. As single cell analytics become more powerful in the study of vascular disorders (42), the ability to procure cells from the intracranial vessels *in vivo* offers a tremendous opportunity for further investigation by the stroke community at large.

In conclusion, we identified a subpopulation of aneurysmal ECs in an altered transcriptional state related to other aneurysm ECs and non-aneurysmal femoral artery ECs in a patient with polyarteritis nodosa. This cell subpopulation demonstrated the enriched expression of hallmark pathways involved in a T-cell mediated immunity and metabolism. Our finding that the immune response pathways are upregulated in these cells and lends support to the notion that the anti-inflammatory and immune-modifying drugs are candidate therapeutics to prevent the intracranial aneurysm formation, growth, and rupture in certain patients.

## Data Availability Statement

The original contributions presented in the study are publicly available. This data can be found here: ArrayExpress under accession number E-MTAB-10681. https://www.ebi.ac.uk/arrayexpress/experiments/E-MTAB-10681/.

## Ethics Statement

The studies involving human participants were reviewed and approved by UCSF Human Research Protection Program (IRB). The patients/participants provided their written informed consent to participate in this study. Written informed consent was obtained from the individual(s) for the publication of any potentially identifiable images or data included in this article.

## Author Contributions

KHN wrote the manuscript and interpreted results. KN, DM, and ZS performed experiments and interpreted results. CH, KM, TT, KC, MA, VH, RH, SH, CD, EW, AA, and TN assisted in interpretation and manuscript revision. DC conceived the project and oversaw the manuscript. All authors contributed to the article and approved the submitted version.

## Funding

We acknowledge funding support from NIH NINDS U54 NS065705 (KHN). The authors also declare that this study received funding from Siemens Healthineers. The funder was not involved in the study design, collection, analysis, interpretation of data, the writing of this article or the decision to submit it for publication.

## Conflict of Interest

The authors declare that the research was conducted in the absence of any commercial or financial relationships that could be construed as a potential conflict of interest.

## Publisher's Note

All claims expressed in this article are solely those of the authors and do not necessarily represent those of their affiliated organizations, or those of the publisher, the editors and the reviewers. Any product that may be evaluated in this article, or claim that may be made by its manufacturer, is not guaranteed or endorsed by the publisher.

## Abbreviations

CTA: computed tomography angiography
MRA: magnetic resonance angiography
FACS: fluorescence-activated cell sorting
scRNA-Seq: single cell RNA sequencing
GSEA: gene set enrichment analysis
DEG: differentially expressed gene
TNFα: tumor necrosis factor alpha
NFκB: nuclear factor kappa-light-chain-enhancer of activated B cells
IFNα: interferon alpha
IFNγ: interferon gamma.
