# Supplementary material for: Endovascular Biopsy of Vertebrobasilar Aneurysm in Patient With Polyarteritis Nodosa
Source: Front Neurol. 2021 Nov 23;12:697105. doi: 10.3389/fneur.2021.697105 (PMC8650719; doi:10.3389/fneur.2021.697105)
Supplement: Supplementary file 1 [file Image_1.PDF]

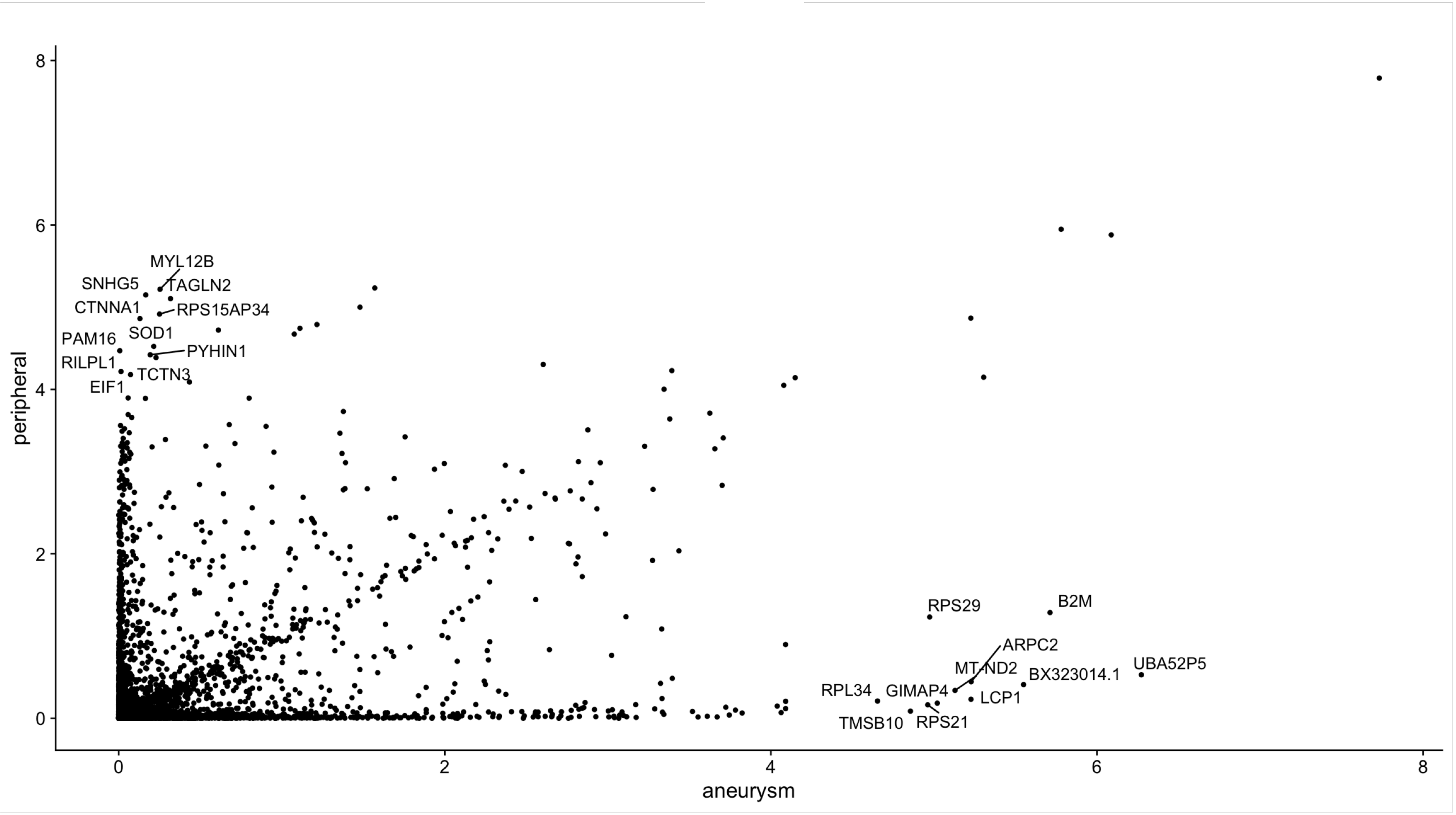

Supplemental Figure 1

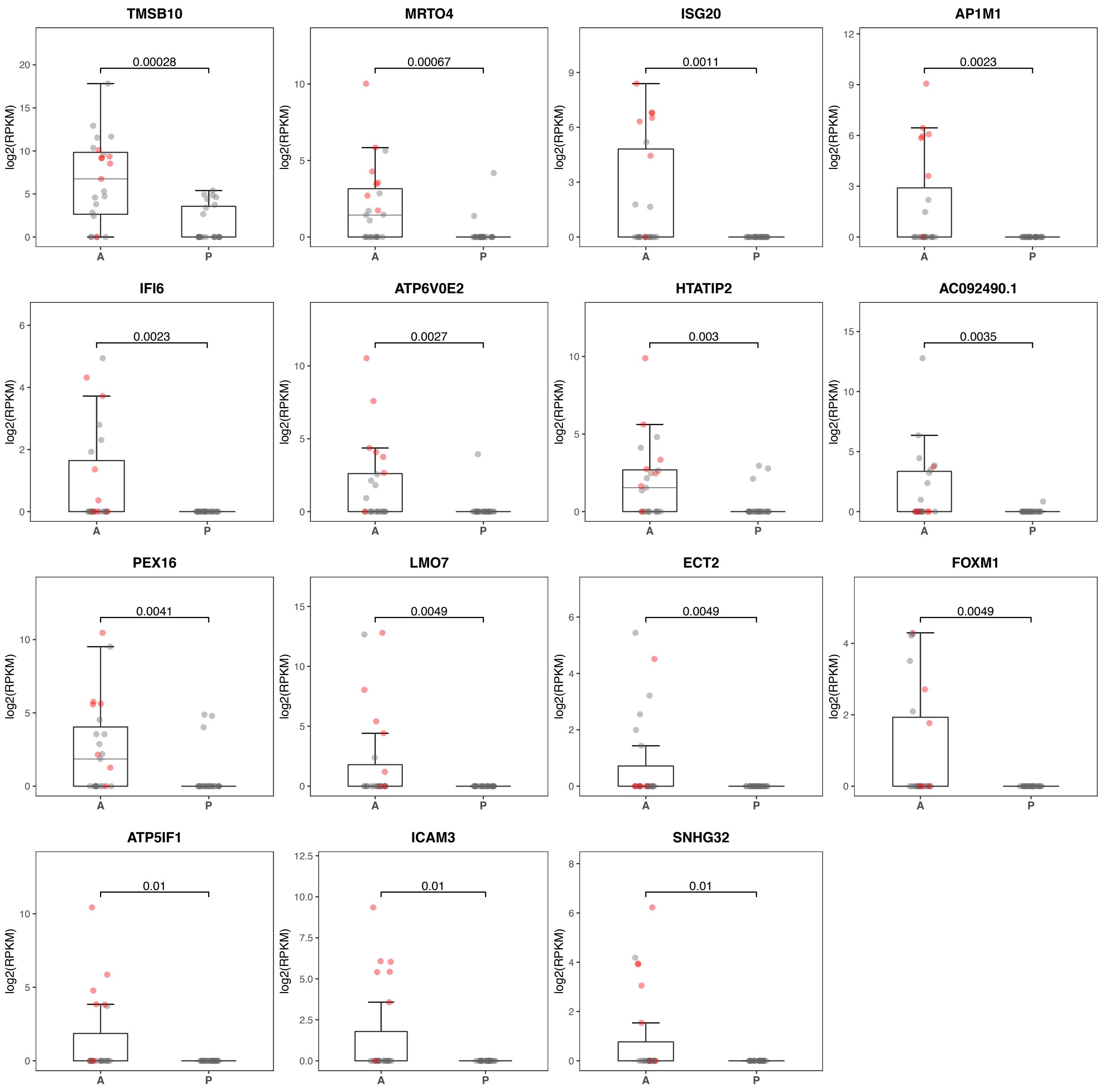

Supplemental Figure 2

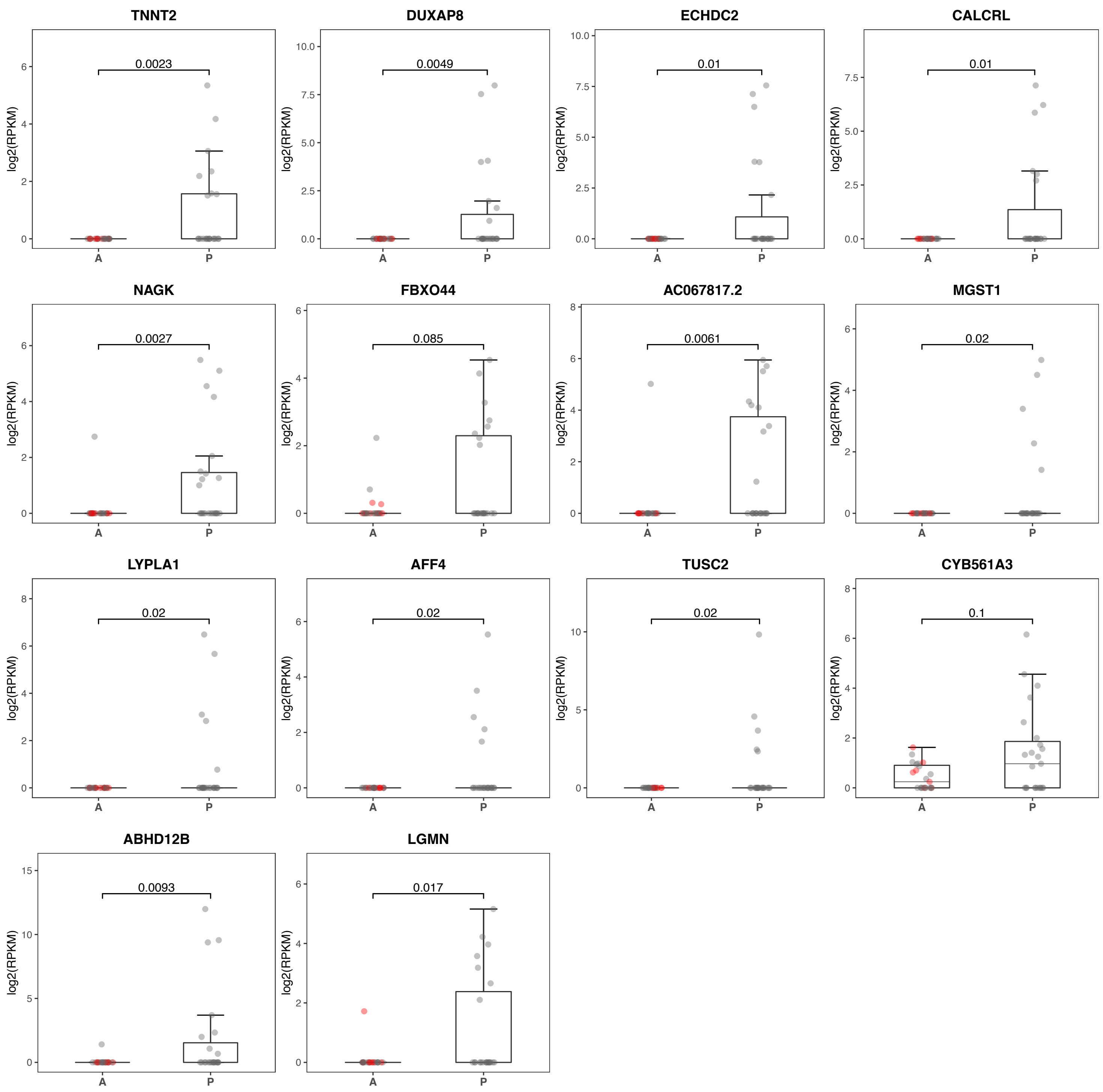

Supplemental Figure 3

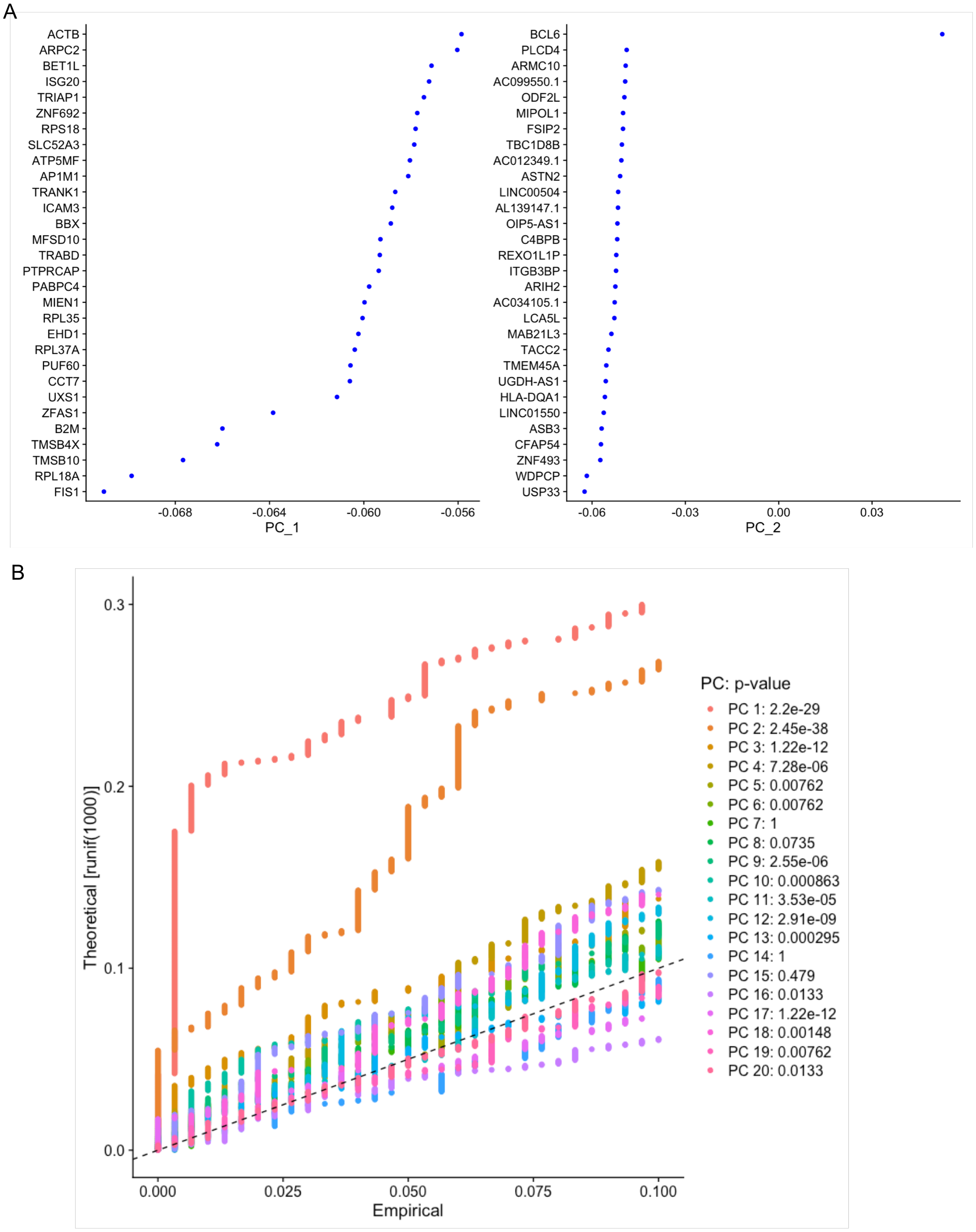

Supplemental Figure 4

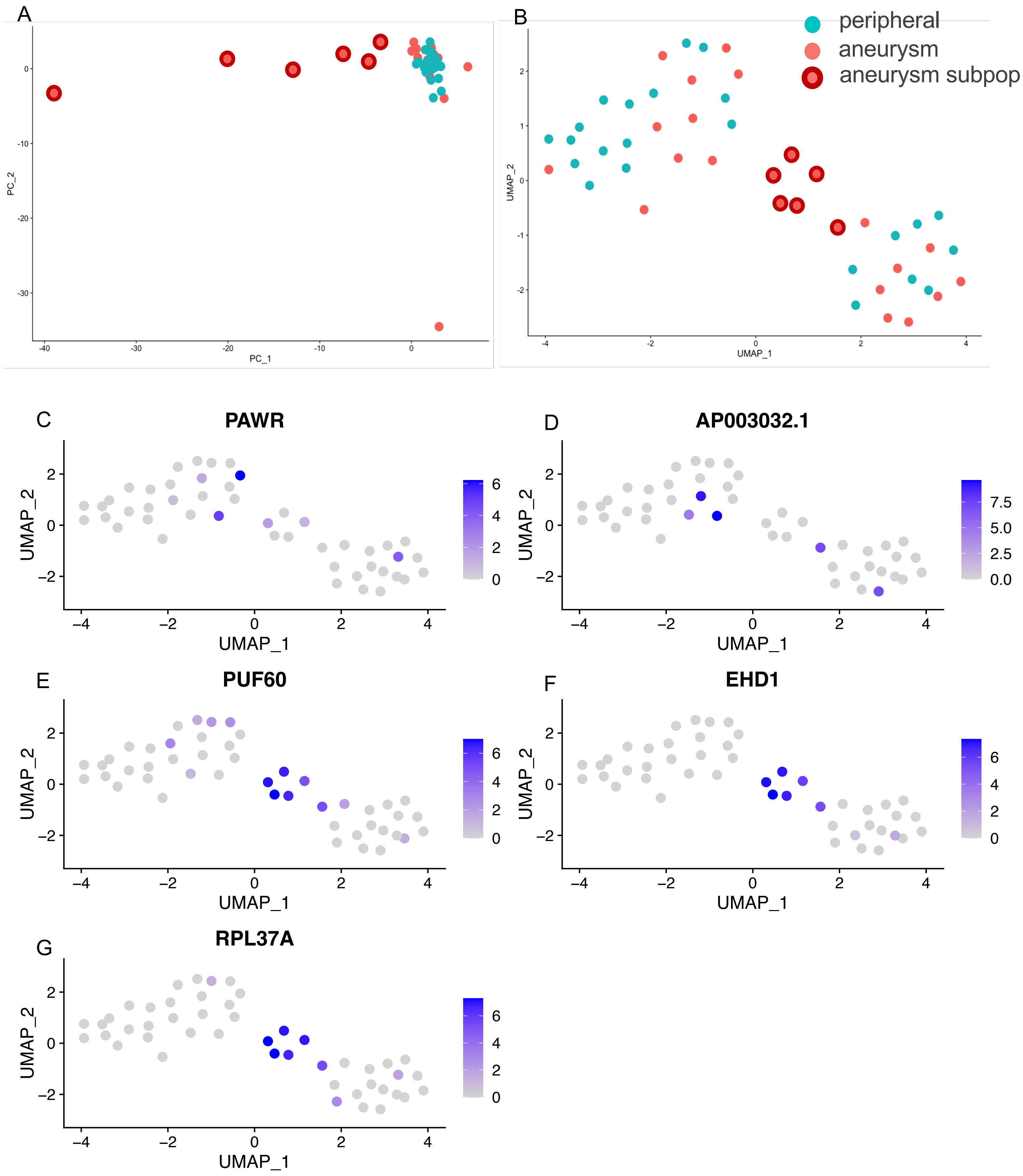

Supplemental Figure 5

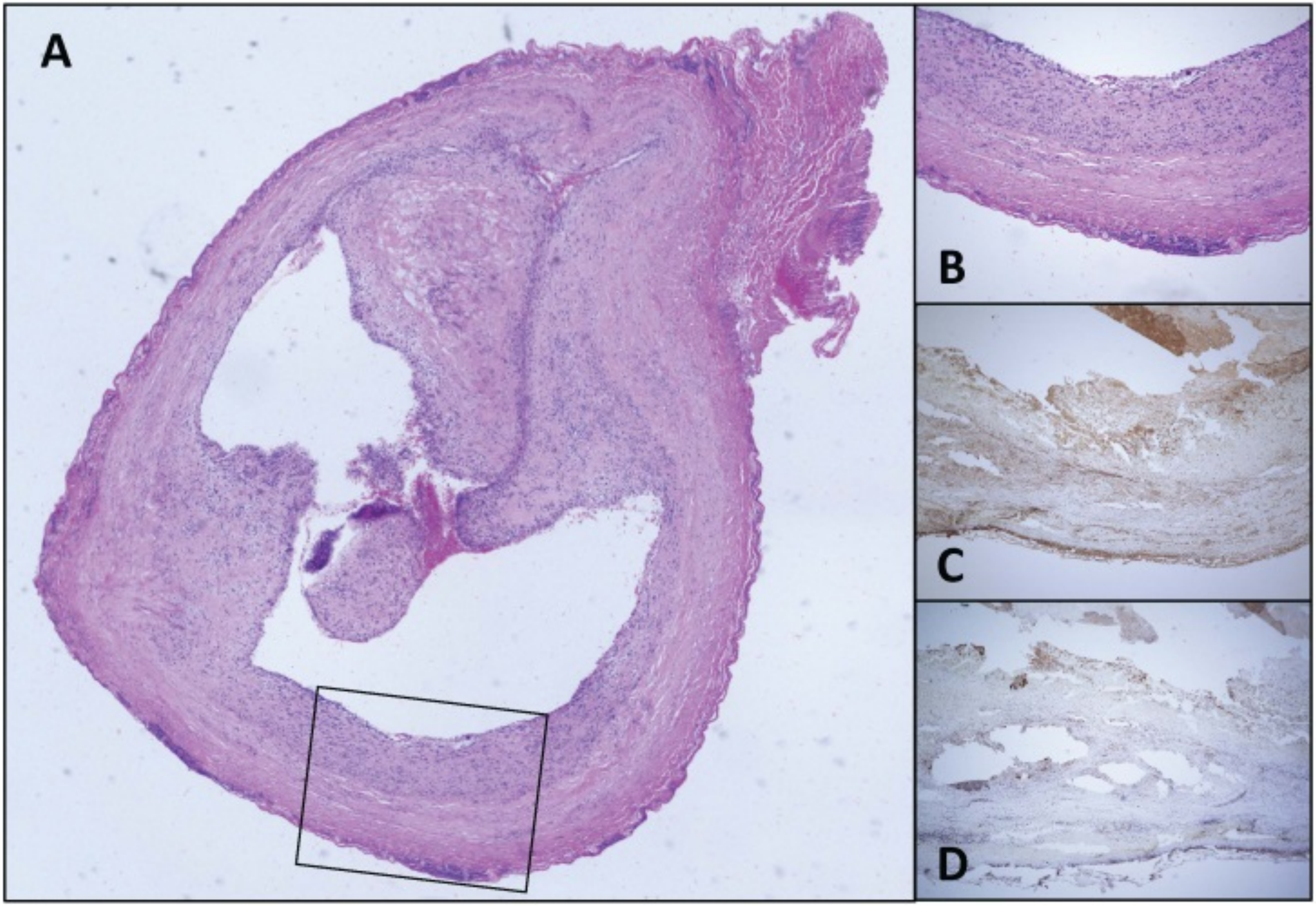

Supplemental Figure 6

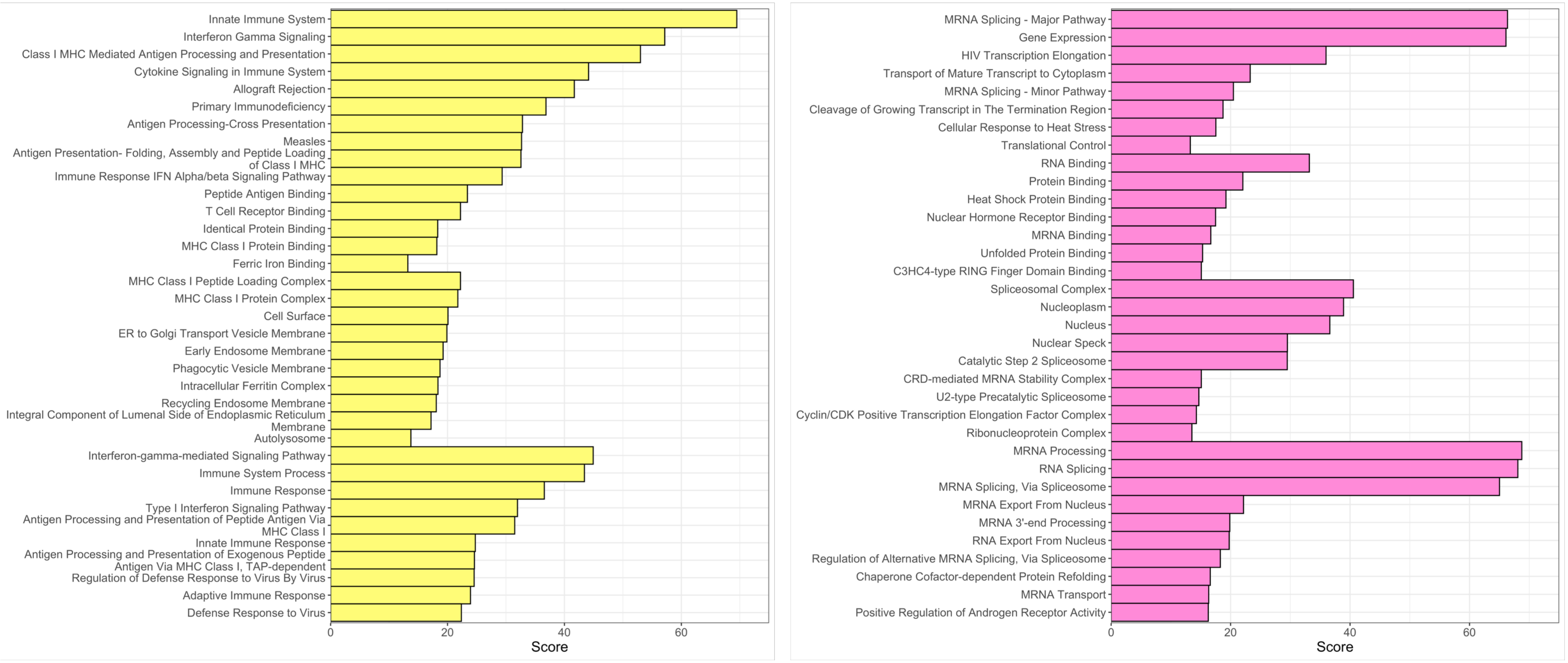

Supplemental Figure 7
