# Supplementary material for: Endovascular Biopsy of Vertebrobasilar Aneurysm in Patient With Polyarteritis Nodosa
Source: Front Neurol. 2021 Nov 23;12:697105. doi: 10.3389/fneur.2021.697105 (PMC8650719; doi:10.3389/fneur.2021.697105)
Supplement: Supplementary file 2 [file Presentation_1.PDF]

Supplemental Methods

Additional Clinical History

A 56-year-old woman with hypertension, hyperlipidemia, and coronary artery disease presented with 3 months of progressive headache, diplopia and dysphagia. Brain MRI/MRA demonstrated vertebrobasilar fusiform aneurysm measuring 31 x 23 x 21 mm on a background of dolichoectasia. The partially thrombosed, fusiform vertebrobasilar aneurysm exerted mass effect on the brainstem. A right posterior inferior cerebellar artery (PICA) aneurysm was also seen measuring 16 x 15 x 12 mm. Subsequent imaging obtained one month later demonstrated growth of the right PICA aneurysm to 19 x 18 x 14 mm. Therefore, treatment of the fusiform vertebrobasilar aneurysm was pursed to prevent further mass effect on the brainstem, while treatment of the right PICA aneurysm was pursued to prevent rupture. Staged treatment was planned, consisting of endovascular flow-diverting stent placement (using Pipeline embolization device) to treat the left vertebrobasilar junction aneurysm, as well as extracranial to intracranial artery bypass surgery (occipital artery to PICA) to enable clip ligation and excision of the PICA aneurysm.

After endovascular flow-diversion and bypass surgery, abdominopelvic CT was obtained to investigate right upper quadrant abdominal pain, demonstrating renal infarcts and multiple additional aneurysms in the splenic, hepatic, and superior mesenteric arteries. Blood tests demonstrated an elevated CRP of 10.9 mg/L and elevated ESR of 60 mm/hr, but tests for hepatitis, HIV, syphillis, tuberculosis, and bacteria were negative. Vasculitis was suspected, and secondary review of the excised PICA aneurysm revealed adventitial and intimal inflammation and focal disruption of the external elastic lamina. Based on her clinical course, systemic and intracranial

arteritis, and tissue pathology, she was diagnosed with polyarteritis nodosa and started on high dose steroids with gradual improvement.

Endovascular biopsy and endothelial cell enrichment. For biopsy of the intracranial aneurysm, a platinum coil attached to a pusher wire (Target 360, Stryker, Salt Lake City, Utah) was advanced through a microcatheter in the right vertebral artery to make contact with the endothelium of the aneurysm at the right vertebrobasilar junction (Figure 1H). The coil did not contact nonaneurysmal endothelium. The coil was placed in dissociation buffer in a 50 ml conical tube, agitated, centrifuged, incubated in erythrocyte lysis buffer, then resuspended in FACS buffer. For the nonaneurysmal peripheral control cells, an 0.035" Bentson guidewire was advanced through a 6 Fr sheath in the right common femoral artery until it made contact with the endothelium of the right common femoral artery and right external iliac artery. After removing the wire from the body, the distal 7 cm was cut and placed in dissociation buffer in a 50 ml conical tube, then processed in the same fashion as the aneurysm sample. After incubation with DAPI, anti-CD31-, and anti-CD34-antibodies, the cell suspensions underwent FACS on an Aria II machine (BD Biosciences, San Jose, California). Cells positive for DAPI were deemed non-viable and excluded. ECs negative for DAPI and double positive for CD31 and CD34 were sorted and individually loaded into a 48-well plate.

scRNAseq

After viable single endothelial cells were dissociated and sorted by fluorescence-activated cell sorting (FACS) on an Aria II machine (BD Biosciences) with viability marker DAPI and endothelial cell specific CD31 and CD34 antibodies, RNA was extracted and libraries were

generated using the Smart-seq2 scRNA-seq protocol on a Fluidigm C1 system and sequenced on the Illumina HiSeq2500 sequencer. cDNA libraries were prepared using the Smart-seq2 protocol on a Fluidigm C1 system (Fluidigm, South San Francisco, California) and sequenced on a HiSeq2500 machine (Illumina, San Diego, California). The Qiagen CLC Genomics Workbench (Qiagen, Redwood City, California) was used for alignment and normalization to calculate read per kilobase per million mapped reads (RPKM). Mapping was performed against human reference genome GRCh37. Then, quality control measures and single cell gene expression profiles were analyzed using the Seurat and Bioconductor packages in R.

Bioinformatic analysis

RPKM-transformed expression values were input to Seurat, a computational pipeline specifically aimed at the analysis of scRNA-seq data. First, QC and internal normalization procedures (using the *NormalizeData* procedure) were performed within Seurat, including the calculation of mitochondrial transcripts (mtRNA) and filtering of cells with >10% mtRNA, which usually represent low-quality cells with broken membranes, for which cytoplasmic RNA will be diminished but RNA within the mitochondria will remain intact. After regressing out biological covariates (ribosomal content and cell cycle effects), a subset of 200 highly variable genes (HVGs) were identified using the *FindVariableFeatures* procedure, and these HVGs were used for dimension reduction using principal component analysis (*RunPCA* procedure) to identify candidate subpopulations within the aneurysm/peripheral cell groups. Complementary to this analysis, the unsupervised clustering algorithm implemented in SC3 was used to disclose cell subpopulations. The optimal number of clusters (*k* parameter) per dataset was estimated using a random matrix theory-based method within SC3 (*sc3_estimate_k* function), and the clustering

algorithm was run using this optimal *k*. Differentially expressed genes (DEGs) between two groups of cell populations/subpopulations were identified using the MAST, Wilcoxon, t-test, ROC, or DESeq2 methods implemented in Seurat (*FindAllMarkers* function). Because the list of DEGs slightly varied depending on the test used, the final list consisted of only DEGs present in all of three test results: Wilcoxon, t-test, and MAST. *P*-values were adjusted for multiple hypothesis testing using a False Discovery Rate (FDR) threshold, and genes were considered differentially expressed when FDR-corrected *P*-value ≤0.05, and log_2-transformed absolute fold-change ≥1. During the exploratory analysis of pathways possibly implicated in the aneurysm phenotypes, nominal *P*-values were used instead, always followed by adjustment of *P*-values estimated from the pathway enrichment analysis.

For enrichment analysis, reference gene sets from MSigDB hallmark processes were used, which are derived by the aggregation of multiple MSigDB gene sets that represent well-defined biological states or processes[8]. The Gene Set Enrichment Analysis (GSEA) framework was run in pre-ranked mode, and an ordered gene list was produced per dataset by scoring each gene according to the following formula:

$$score = -log_{10}(P) \times sign(FC),$$

where *P* is the *P*-value estimated from the differential expression analysis, and FC is the corresponding fold-change value derived from comparisons between two groups of cell populations. One thousand permutations of the gene sets were performed to calculate gene-set FDR, and a cutoff of 0.05 was used to define significance. During the exploratory analysis of pathways possibly implicated in the aneurysm phenotypes, nominal *P*-values were used, always followed by adjustment of *P*-values estimated from the pathway enrichment analysis. One

thousand permutations of the gene sets were performed to calculate gene-set FDR, and a cutoff of 0.05 was used to define significantly enriched pathways. Only biological process terms from the Gene Ontology (GO) were considered, and GO terms were grouped when their kappa score was ≥0.4. Visualizations were produced in R (http://www.r-project.org/) using base functions and the packages ggplot2, circlize, pheatmap (https://github.com/raivokolde/pheatmap), and clueGO.

For network analysis, differential gene expression analysis was performed on the subpopulation of aneurysmal endothelial cells using MAST. These DEGs were fed into the STRING database in order to retrieve known protein-protein interactions. A network was constructed where the nodes represent proteins and the edges represent an interaction between any two proteins. Node color represents average log FC in expression, such that darker red colors signify a large positive fold change in expression, and darker blue colors signify a large negative fold change in expression. Node size corresponds to the p-value of the DEG. Clustering was then performed using the MCODE (Molecular Complex Detection) algorithm, which detects highly interconnected regions in the network. This clustering demonstrated a cluster of nodes related to the immune system and a cluster of nodes related to metabolism (Figure 3A). Pathway enrichment analysis was then performed in the member nodes of the cluster (Supplemental Figure 7). The DEGs were then imported into ClueGO, a Cytoscape plug-in to visualize functionally grouped terms as networks to facilitate biologic interpretation.

Supplemental Figure Legends

Supplemental Figure I. Scatter plot of average expression levels in aneurysmal and peripheral endothelial cells. Gene expression in all 24 aneurysm ECs were averaged and plotted on the

horizontal axis, while average gene expression in all 23 peripheral ECs was plotted on the vertical axis. Visual outliers are highlighted with gene names.

Supplemental Figure II. Box-whisker plots of differentially expressed genes (DEGs) that were overexpressed in the aneurysmal versus the peripheral endothelial cells and consistently identified by three methods: MAST, Wilcoxon, and t-test. Unadjusted p-values are shown.

Supplemental Figure III. Box-whisker plots of differentially expressed genes (DEGs) that were overexpressed in the peripheral versus the aneurysmal endothelial cells and consistently identified by three methods: MAST, Wilcoxon, and t-test. Unadjusted p-values are shown.

Supplemental Figure IV. (A) Features (genes) that define the first two principal components are shown. (B) Jack-Straw plot comparing the distribution of p-values for each principal component with a uniform distribution (dashed line).

Supplemental Figure V. (A) Principal component analysis (PCA) and (B) uniform manifold approximation and projection (UMAP) analysis of single cell RNA sequencing results demonstrates a subpopulation of aneurysmal endothelial cells (orange with red border) in altered transcriptional state relative to the other aneurysmal endothelial cells (orange) and nonaneurysmal peripheral femoral endothelial cells (turquoise). (C-G) Expression heatmaps for select differentially expressed genes (DEGs) shows that they are enriched in the subpopulation.

Supplemental Figure VI. Histologic evaluation of a separate aneurysm in the same patient excised from the left posterior inferior cerebellar artery (PICA). (A) Cross-section of the aneurysm dome at 4x magnification. (B) Diffuse inflammation in the aneurysm wall at 10x magnification. Immunohistochemical stain for NFkB (C) and TNFa (D) demonstrate strong positive staining (brown).

Supplemental Figure VII. Protein-protein interaction network to identify signal transduction pathways of interest in the aneurysm endothelial cell subpopulation. Differentially expressed genes (DEGs) between the aneurysm subpopulation and the remaining cells were identified using MAST, then fed into the STRING database (see Supplemental Methods). The resulting network contains 219 nodes with 630 edges. Clustering using the MCODE algorithm detects highly interconnected regions of the network. An immune system subnetwork is highlighted in yellow and a metabolism subnetwork is highlighted in pink (Supplemental Figure 8). Node color represents average logarithmic fold change and node size corresponds to p-value (see legend at right).

Supplemental Figure VIII. Subnetworks of the protein-protein interaction network shown in Figure 3 are enriched in immune system (yellow; left) and metabolism (pink, right) pathways.

Supplemental Table I. Differentially expressed genes in the aneurysmal endothelial cells compared to the peripheral endothelial cells using MAST. None of the p-values met criteria for statistical significance after multiple testing correction.

Supplemental Table II. Differentially expressed genes in the subpopulation of aneurysmal endothelial cells compared to the remaining endothelial cells using DESeq2. 101 genes met p-value criteria for statistical significance after multiple testing correction.
